# Barriers to provision of respectful maternity care in Zambia: results from a qualitative study through the lens of behavioral science

**DOI:** 10.1186/s12884-019-2579-x

**Published:** 2020-01-09

**Authors:** Jana Smith, Rachel Banay, Emily Zimmerman, Vivien Caetano, Maurice Musheke, Ameck Kamanga

**Affiliations:** 1 ideas42, New York City, USA; 2 Population Council, Lusaka, Zambia; 3 Jhpiego, Lusaka, Zambia

**Keywords:** Respectful maternity care, Qualitative, Zambia, Disrespect and abuse, Providers, Behavioral science, Behavioral economics, Provider behavior change, Experience of care, Maternal care

## Abstract

**Background:**

Recently, a growing body of literature has established that disrespect and abuse during delivery is prevalent around the world. This complex issue has not been well studied through the lens of behavioral science, which could shed light on the psychological dimensions of health worker behavior and how their micro-level context may be triggering abuse. Our research focuses on the behavioral drivers of disrespect and abuse in Zambia to develop solutions with health workers and women that improve the experience of care during delivery.

**Methods:**

A qualitative study based on the behavioral design methodology was conducted in Chipata District, Eastern Province. Study participants included postpartum women, providers (staff who attend deliveries), supervisors and mentors, health volunteers, and birth companions. Observations were conducted of client-provider interactions on labor wards at two urban health centers and a district hospital. In-depth interviews were audio recorded and English interpretation from these recordings was transcribed verbatim. Data was analyzed using thematic analysis and findings were synthesized following the behavioral design methodology.

**Results:**

Five key behavioral barriers were identified: 1) providers do not consider the decision to provide respectful care because they believe they are doing what they are expected to do, 2) providers do not consider the decision to provide respectful care explicitly since abuse and violence are normalized and therefore the default, 3) providers may decide that the costs of providing respectful care outweigh the gains, 4) providers believe they do not need to provide respectful care, and 5) providers may change their mind about the quality of care they will provide when they believe that disrespectful care will assist their clinical objectives. We identified features of providers’ context – the environment in which they live and work, and their past experiences – which contribute to each barrier, including supervisory systems, visual cues, social constructs, clinical processes, and other features.

**Conclusions:**

Client experience of disrespectful care during labor and delivery in Chipata, Zambia is prevalent. Providers experience several behavioral barriers to providing respectful maternity care. Each of these barriers is triggered by one or more addressable features in a provider’s environment. By applying the behavioral design methodology to the challenge of respectful maternity care, we have identified specific and concrete contextual cues that targeted solutions could address in order to facilitate respectful maternity care.

## Background

A growing body of literature has established that disrespect and abuse during delivery is prevalent in settings around the world, and not only affects the quality of the delivery and postnatal experience itself, but influences subsequent interactions with the healthcare system. For instance, those with positive delivery experiences are more likely to deliver in a facility for a subsequent birth [1], and those with a preference for skilled providers during maternity care are more likely to attend postnatal care [2, 3].

Studies on this topic have documented and described the problem of disrespectful care and abuse during facility-based birth in both higher and lower-income settings [4]. A recent review suggested that prevalence of disrespect and abuse may fall between 15 and 98%, and can include issues from poor provider-client rapport to physical and sexual abuse [5].

With evolving taxonomies and a proliferation of localized studies, a standardized measure of the prevalence of disrespect and abuse has not emerged or been applied universally. In Zambia, research on respectful maternity care has been limited, but there is suggestive evidence that providers frequently fail to provide respectful care during facility-based delivery [6]. For instance, in a qualitative study, women in Kalomo district in Zambia described being shouted at or abandoned during the labor process [6] in a way that deterred some from delivering in a facility in subsequent pregnancies [9].

Respectful Maternity Care Definition

**Table:** 

According to the World Health Organization, respectful maternity care is “care organized for and provided to all women in a manner that maintains their dignity, privacy and confidentiality, ensures freedom from harm and mistreatment, and enables informed choice and continuous support during labor and childbirth.” [8] Examples of respectful care include, but are not limited to, allowing women to make decisions about their care such as whether they would like a companion, asking permission to conduct procedures and explaining those procedures to women, and ensuring women know their rights. The Respectful Maternity Care Charter outlines rights of childbearing women [9], which respectful care upholds.	

While research has sought to understand the drivers of this complex issue [10–12], respectful maternity care has only recently begun to be studied through the lens of behavioral science globally [13, 14]. Bringing the insights of behavioral science to bear on respectful maternity care can shed light on the psychological dimensions of provider behavior, such as the instinct to justify disrespectful care and perceptions of what respectful care encompasses, and identify how providers’ micro-level context could trigger disrespect and abuse. That context can range from lessons during midwifery school and dynamics across providers within one facility, to individual interactions with mothers. Identifying granular features of providers’ context that could trigger disrespect and abuse affords opportunities to address these issues through innovative design, and ultimately improve the experience of care during delivery.

Our study addresses gaps in the literature by applying a behavioral science lens to identify features of the local context that drive disrespectful and abusive care, and by documenting the experience of disrespectful and abusive care in Zambia, where previous work on this topic has been limited. Because consequences of disrespectful maternity care like stress and fear can lead to poor birth outcomes [15] and delay lactation [16], and disrespectful and abusive experiences can reduce clients’ willingness to access health services in the future [17], improving the quality of facility-based birth by uncovering and addressing the behavioral drivers of providers’ care has the potential to impact many lives, both of mothers and infants.

The aim of the study was to understand the behavioral drivers of disrespect and abuse during labor and delivery in Zambia, in order to develop solutions together with health workers and women to improve the experience of care during delivery.

## Methods

### Setting and participants

We conducted fieldwork in Chipata district, Eastern Province, Zambia during 2 weeks in July, 2018. An urban area, Chipata has the largest population, and population density, of Eastern Province [18], as well as a number of health facilities with high client volumes. We selected Chipata as a fieldwork site due to providers’ lower compliance with clinical best practices in the district and upon recommendation of our local partner organization. To understand the drivers of disrespect and abuse, we undertook a cross-sectional qualitative research study comprising in-depth interviews with key stakeholders and observations of interactions on the labor ward. We conducted interviews with parties with different perspectives on provider-client interactions during labor and delivery: providers, their supervisors, birth companions, postpartum women who had given birth in the last 6 months, and Safe Motherhood Action Group (SMAG) volunteers. SMAG volunteers are community members who liaise between health facilities and clients in the community, often conducting outreach activities. These perspectives were chosen to capture the range of experiences from those involved with providing or overseeing service delivery to those giving birth or accompanying a woman giving birth. We determined sample sizes based on the likely diversity of experience (higher among individual clients while relatively lower among providers in similar facilities) as well as expectations of saturation. These interviews were conducted in two rural health centers, two urban health centers, and one central hospital, selected to represent a range of experiences in the region. We also conducted several multi-hour observations of clinical care and provider-client interactions on labor wards at two urban health centers and a central hospital (no deliveries coincided with our visits to rural health centers) until we had observed a wide range of providers, delivery circumstances, and interpersonal dynamics.

The two urban health centers where we conducted interviews and observations offer a range of services such as in- and out-patient services, family planning, voluntary counseling and testing, and under 5 clinics, and are both located in Eastern Chipata. They experience a high client volume such that our research visits over 2 weeks typically coincided with at least one delivery. The two rural health centers where we conducted interviews offered a similar range of services and experienced much lower client volume; we did not intersect with a delivery during several visits over 2 weeks. The central hospital is one of two general hospitals in Eastern Province [18], and regularly experiences very high client volumes.

### Theoretical framework

Our first step in developing the research tools was to draw up a detailed process map of both clinical and interpersonal steps generally considered as best practice in routine deliveries. For clinical steps, we used the version of the Safe Childbirth Checklist [17] which had been adapted to Zambia, and for interpersonal pieces we drew from the Respectful Maternity Care Charter [8] as well as research tools used by the Averting Maternal Death and Disability project. We then solicited feedback from a local clinician to ensure that this list of discrete behaviors was up to date with Zambian delivery practice. After finalizing the process map, we then used a series of organizationally-developed question prompts, which cover different psychological principles identified in a wide range of behavioral science papers [19–22], to generate a list of hypotheses as to the potential behavioral barriers inhibiting providers from providing quality respectful care and the factors in the environment that might precipitate these barriers. We also drew from published literature on barriers to respectful maternity care in other settings to generate additional hypotheses to explore in our fieldwork. Each hypothesis consisted of a behavioral barrier and one or more features in the context of the provider which could be triggering the barrier. Using the hypothesized barriers and contextual factors as a basis, we generated interview guides to structure conversations with participants, and observation guides to structure observations of clinical interactions.

### Materials and processes

In Chipata, a contact from our local partner organization that provides supportive supervision to maternal and child health providers in local facilities made introductions to staff at two urban and two rural health facilities. We obtained permission from facility supervisors to speak with and observe their staff, and conducted an informed written consent process with each participant before beginning the interview. Participants received 50 kwacha (approximately $5 USD) as compensation for their time.

For interviews with providers and supervisors, we invited maternal and child health and labor ward staff to participate in interviews at the facilities they work in at times when they were not otherwise occupied with professional obligations. Interviews with providers concerned participant background, professional responsibilities, typical client behaviors and providers’ responses to these behaviors, and elements of respectful care. Interviews with supervisors concerned participant background, supervisory responsibilities, provider practices, and typical client behaviors.

For interviews with clients, birth companions and SMAGs, interviews took place in the facility itself or in clients’ homes (SMAGs helped introduce us to women in the community who had recently given birth, or to their companions). Interviews with clients and birth companions concerned participant background, preparation for delivery, the client’s birth experience, provider-client dynamics, and positive and negative elements of the experience. Interviews with SMAGs concerned participant background, volunteer responsibilities, perceptions of providers and clients, typical client behaviors, and providers’ responses to these behaviors.

We conducted observations of the labor ward when clients were in labor or delivery, provided that clients and providers felt comfortable with our presence and completed an informed consent process.^1^ Observations were captured in as objective and detailed a fashion as possible including all conversations and interactions between client and provider and any other parties present, as well as all the provider’s clinical activities and the manner in which they were conducted.

We obtained ethical approval from a U.S.-based as well as a local Institutional Review Board to whom we submitted detailed interview guides and observation protocols. We obtained written informed consent from each interview and observation participant before beginning interviews and observations, and discussed with participants that they were under no obligation to participate, could discontinue at any time, and could decline to answer any question they chose.

### Analysis

In-depth interviews were audio recorded and English interpretation from these recordings was transcribed verbatim. Observation notes were transcribed for a comprehensive log of events and interactions. We employed thematic analysis, drawing from the process outlined by Braun and Clarke [23]. An initial set of codes was drawn from the hypothesized barriers and contextual factors described above, and additional codes were added during the coding process as new insights emerged. Data were coded by members of the research team. Several observation logs and transcripts were double-coded to ensure consistency. Coded data were matched to each relevant hypothesized barrier and contextual feature, and members of the research team individually assessed the extent to which the evidence supported or contradicted the hypothesis, or whether evidence was mixed. These assessments included a consideration of how plausible a link was established between confirmed contextual factors and barriers either through direct evidence that was coded or other behavioral literature on how contextual factors commonly shape subconscious decision-making. Individual assessments were compared and any discrepancies were resolved through discussion until consensus was reached regarding the barriers that the evidence most clearly indicated to play a significant role in client disrespect and abuse, based both on the volume and strength of evidence.

This manuscript follows the O’Brien et al. Standards for Reporting Qualitative Research [24].

## Results

### We conducted 46 individual interviews and nine multi-hour observations at health facilities (see Table 1)

**Table 1:** Summary of Individual Interviews and Respondent

Characteristics Participant type	Number of participants	Respondent characteristics
Total	46	
Provider	17	Interview locations:
		Central hospital: 3
		Urban clinic: 11
		Rural clinic: 3
		Gender of respondents:
		15 female
		2 male
Midwife supervisor or mentor	3	Interview locations:
		Urban clinic: 1
		Rural clinic: 2
		Gender of respondents:
		1 female
		2 male
SMAG volunteer	7	Interview locations:
		Urban clinic: 4
		Rural clinic: 3
		Gender of respondents:
		5 female
		2 male
Client	15	Interview locations:
		Urban clinic: 4
		Rural clinic: 3
		Community setting: 8
		Gender of respondents:
		15 female
Birth companion	4	Interview locations:
		Community setting (urban): 4
		Gender of respondents:
		4 female

We identified five key behavioral barriers inhibiting respectful maternity care: 1) providers do not consider the decision to provide respectful care because they believe they are doing what they are expected to do, 2) providers do not consider the decision to provide respectful care explicitly since abuse and violence are normalized and therefore the default, 3) providers decide that the costs of providing respectful care outweigh the gains, 4) providers believe they do not need to provide respectful care, and 5) providers change their mind about the quality of care they will provide when they believe that disrespectful care will assist their clinical objectives. We identified specific features of providers’ context – the environment in which they live and work and their past experiences – which contribute to each barrier. Table 2 summarizes these associated contextual features and the level at which they occur.

**Table 2:** Summary of Barriers and Contextual Features at the Individual, Interpersonal, Organizational, and Community Levels

Barriers to Respectful Maternity Care	Contextual Features			
	Individual level	Interpersonal level	Organizational level	Community level
▪ Providers do not consider the decision to provide respectful care because they already believe they are providing respectful care or what they are expected to do	▪ Provider had a painful delivery and has attended many painful deliveries ▪ With experience provider has developed a “feel” for how care is provided	▪ Supervision and feedback focused on clinical treatment and health risks	▪ Training is focused on clinical treatment ▪ Clinical algorithms and guidelines, including visual cues in the facility do not provide clear guidelines for good care	▪ Pain is seen as a natural birth experience
▪ Providers do not consider the decision to provide respectful care explicitly since abuse and violence are normalized and therefore the default	▪ Provider experienced violence as a child as a form of discipline	▪ Actions of other providers reinforce the perception that maintaining control is paramount	▪ Training emphasizes need for rigid, forcefully delivered commands and interventions	
▪ Providers decide not to provide respectful care since they believe they do not need to provide it		▪ Provider has never interacted with the client before delivery and client behaves erratically or does not follow instructions	▪ No serious consequences to providers who engage in disrespectful or abusive behavior	▪ Client appears to be low income or low status
▪ Providers decide not to provide respectful care consistently since they believe that the costs of providing it outweigh the gains			▪ Maternal or infant death results in an audit ▪ No salient information or feedback on the impact of respectful or disrespectful care on health outcomes	
▪ Providers change their mind on providing respectful care when they believe that disrespectful care will assist their objectives		▪ Client does not follow instructions of provider		

It should be noted that not all barriers apply to all providers in all circumstances. Instead, these barriers describe the range of factors which may explain why a particular provider may never solidify an intention to provide respectful care or may intend to provide respectful care but act in a way which is not aligned with that intention.

### Barrier 1. Providers do not consider the decision to provide respectful care because they already believe they are providing respectful care or what they are expected to do

The providers we interviewed and observed generally reported that they were fulfilling the expectations of their role which centered on clinical aims. Our research highlighted four features in their context which likely shape their understanding of what is expected of them in ways which also inhibit reflection around the degree to which they are adhering to practices related to respectful care.

#### Training, supervision and feedback is focused on clinical treatment and health risks and does not address respectful care

Through observation and interviews with providers and supervisors, it became clear that training, supervision and feedback providers receive centers on the clinical aspects of their role and does not include most elements of respectful care. Providers described success in their role in terms of survival of the client and her child thus implying that providers may tunnel on these clinical outcomes without considering other elements of care as explicitly [19].

#### There are clinical algorithms and guidelines, including visual cues in the facility, but nothing which provides clear guidelines for how to give good care

Providers’ understanding of the clinical aspects of their role was concrete and reinforced visually throughout the facility. For example, facility walls were often plastered with posters outlining steps to take to prevent poor clinical outcomes, such as if a baby was not breathing. Similarly, providers used the partograph to identify when a delivery was at risk of complications. By contrast, our observations revealed that there were no clear guidelines or visual cues for whether, when, and how to provide respectful care. When asked about respectful care, providers generally described care absent of bias or stated a lack of familiarity with the term.

#### Pain is seen as a natural birth experience — the provider had a painful delivery, has attended many painful deliveries, and the bible says that labor is painful

Interviews with providers around pain management and observation of deliveries suggested that most providers viewed pain as an unavoidable part of the process. When asked about options for pain relief, responses varied from talking to the client to calm her to noting that nothing could be done since pharmacological pain relief was not available. Respondents spoke about personal experience delivering, professional experience in which painful deliveries are the norm, and scripture and religious messaging on the pain inherent to childbirth, without remarking on what they do could to support clients. Clients noted this lack of empathy or alleviation acutely: “they should comfort us at least, when you are in pain. Just telling you, ‘it’s okay, you’ll be fine,’ you know you feel okay then … It feels bad when you’re in pain and then someone is adding another pressure on you.”

#### Provider has attended many deliveries and developed a “feel” for how care is provided

Experienced providers often described having developed a “feel” or instinct for what must happen during delivery. For example, one provider compared it to cooking: “Once you learn for the first time, you don’t forget how to cook … You already know that I will do this, I will do this, I will do this.”

### Barrier 2. Providers do not consider the decision to provide respectful care explicitly since abuse and violence are normalized and therefore the default

Normalization of violence, driven by the contextual features described below, positions scolding, yelling, or slapping prominently in the choice set of providers as a means to get clients to comply with requests. Other barriers explain why client compliance may appear critical to providers, but this barrier helps to explain why providers may not even consider how clients or others might perceive their behavior as disrespectful or poor care.

#### Provider experienced violence as a child as a form of discipline

Providers commonly reported that as children they were yelled at or slapped when they did something they were not supposed to do. As one provider recollected, “I think when I went wrong, I was slapped by the parents, it’s normal, it was part of maybe disciplining me.” Providers did not mention that this strategy was inappropriate or detail how times may have changed.

#### Training and clinical experience of provider reinforces that clients need rigid, forcefully delivered commands and interventions

Providers in training remarked that during formal education, they are often taught that “good” care goes beyond complying with clinical protocol and keeping clients safe to include client support and interpersonal elements of care. However, once they arrive at a clinic or hospital as part of their on-site training, providers remarked that they were taught by other providers that rigid commands are often necessary to get clients to comply. One student midwife reflected on how her clinical experience had changed her views of what was necessary, noting that “some they will say, ‘no, midwives are bad, midwives shout at us’ … when I was not in the health sector I was thinking like that too, but when I came here...no wonder you see they are very strict ... because they know there might be harm [from] some of the things that you want to do.” Several midwives in training noted that they did not agree with this way of providing care but that it is difficult for new providers to counter this norm. As one student midwife noted “you’re just a student and them, they have experience there, so if you tell them ‘no, madam, listen, listen,’ some may feel as if you’ve just underrated them.”

### Barrier 3. Providers decide not to provide respectful care since they believe they do not need to provide it

Providers may consciously or subconsciously consider the decision of providing elements of respectful care and make a decision that they do not need to provide it. The contextual features driving this decisional barrier influences both what a provider perceives to be necessary and what she perceives a client to deserve.

#### There are no serious consequences to providers who engage in disrespectful or abusive behavior

Interviews implied that many providers who actively disrespected clients experienced no consequences or only minor consequences as a result. One provider, when asked what happens to a provider if she hits a client, remarked, “we apologize to the patient, ‘please forgive me,’ and that’s all you do.” Providers who see the lack of consequences learn that they do not need to provide respectful care to remain in good standing in their jobs. One postpartum woman’s experience illustrates this clearly: her mother threatened to tell the provider’s supervisor that the provider had told her to mop the floor. The provider responded “Go and report, I don’t care. Are you the one who employed me?”

It is interesting to note that there seems to be variation in the prevalence of this feature between rural and urban contexts. For instance, in certain rural areas complaints about disrespectful care were received by the village chief. According to one client, the chief’s intervention reduced incidents of disrespectful care: “things like [yelling at or slapping the client] used to happen at this clinic, but everyone who did that was given a forced transfer by the chief.” On the other hand, formal complaints about disrespectful care in urban settings were made to the in-charge or through a suggestion box, which according to clients did not lead to change. One postpartum woman noted that “even if [people use the suggestion box], they will never publicize it, so it’s like they hide when something bad is written on it.”

#### Client clothing or appearance makes them seem low-income, or they are considered to be a community member of lower status

While the providers we interviewed reported that they provided equal care to all, remarks from other respondents suggest that this is often not the case, especially in urban areas where income inequality is more common. Many respondents, including clinical mentors, post-partum women, and even providers noted that clients who were perceived as wealthy were treated with more kindness than clients who were perceived as low-income. Income status was judged by the car clients arrived in as well as the quality of the bag and materials they brought with them to the facility. When asked about this, one client reflected: “I don’t know [why poor clients are treated worse], it’s only [providers] who know. Just from the appearance [they know who is poor], you know they say, ‘give us your bag,’ [they check] what’s in the bag.”

When a client is wealthy or from a family of status or power, providers believe that these individuals have the power to get them fired and therefore they feel that they must provide good care. One supervisor explained: “a patient who is very poor won’t be treated well, just because that person cannot offer anything or cannot do anything. If I am dressed well I will be attended to.” When attending clients perceived as low status, providers may believe that these clients have no voice or power and as such they do not need to provide them with good care.

Additionally, general societal stigma towards lower-income people leads providers to believe that they are not deserving of care. Several providers noted their “white uniform” or a desire not to dirty their uniform, which seemed to be a feature that contributed to their identity as a provider. For example, we observed a few providers telling another provider to stop cleaning a client who had just given birth since this would “get her apron dirty.” One respondent further emphasized the status associated with the white uniform when describing her aspirations to become a provider. Providers also noted the presence of bodily fluids in their daily work, and some described a visceral reaction to these fluids. One provider noted it was her least favorite part of the job: “I [never] get used to them.” Providers may be even more sensitive towards “dirtying” their uniforms among lower-income people they believe to be less clean, as this uniform serves as a visible differentiator in status between providers and clients and a form of pride for certain providers.

#### Provider has never interacted with the client before delivery and the client is behaving erratically or not following instructions

Antenatal care and delivery care are administered in separate wards and providers reported that they meet clients for the first time when they arrive to the facility to deliver. During labor, we often observed clients crying out, “Mommy, mommy,” while writhing on the hospital bed. Observation suggested that providers may view this behavior as similar to that of children which may affect how they view the client; providers are unlikely to believe that children need to consent to procedures or receive explanations for what is occurring. Furthermore, given providers’ experiences as children being scolded or slapped described previously, being harsh may be the default behavior to correct bad behavior among children.

Providers often attributed lack of client cooperation to a woman’s personality rather than her situation in labor which suggests that attribution error may also impact provider behavior [25]. One provider explained, “Sometimes they are just like that, difficult in labor...they behave as if they are mad. Some don’t cooperate.” Observation and client interviews suggest that when a client does not cooperate, many providers believe it is the client’s fault and that a difficult client does not deserve good care.

### Barrier 4. Providers decide not to provide respectful care consistently since they believe that the costs of providing it outweigh the gains

Providers consistently reported focusing on avoiding death of the client and her child, and defined success as both of them surviving. Their remarks highlighted that the risk that the client or her child could die in childbirth looms large in providers’ minds, and that their attention and energy is focused on keeping them alive by all means necessary. As several providers reflected, the “most important thing is to deliver a live baby. When they go into second stage, you have to be very vigilant.” Another added, “there are two things [that are the worst for a provider]: maybe maternal death, then we have stillbirth.” With an often singular focus on the goal of keeping the client and her child alive, our observation and interviews highlighted that some providers resort to tactics that involve disrespectful care such as shouting at, hitting, or refusing to attend clients who do not follow directions.

#### Maternal or infant death results in an audit, placing an emphasis on clinical practices

Providers rightly anticipate that if a client or her child dies, there will be an investigation into the causes of death, and potential consequences for the provider for any mistakes she made. As one provider reflected, “If we have maybe a stillbirth, they’ll still come back to the reasons; what led that baby to die … was it negligence or what?” This audit reinforces the emphasis on clinical practices present throughout the physical environment and the provider’s training and supervision.

#### Providers do not receive salient information or feedback on the impact of respectful or disrespectful care on health outcomes

While the link between clinical practices and health outcomes is clear to providers, the impact of respectful or disrespectful care is less clearly understood. Although providers occasionally remarked that keeping clients calm during labor has benefits for the baby, they never articulated all of the potential health consequences of disrespectful care. Furthermore, when providers shared experiences from their training and feedback they did not mention any discussion of the connections between respectful care and health outcomes, therefore implying that these links are not clear.

### Barrier 5. Providers change their mind on providing respectful care when they believe that disrespectful care will assist their objectives

While many providers spoke about wanting to provide respectful care, other comments suggest that they may ultimately change their minds and provide care which is harsh or abusive. Providers often highlighted the risky nature of the work they do and how any adverse development could become extreme and lead to the death of a client or a child. Providers perceive the client’s own actions as instrumental in averting disaster; therefore, providers often spoke of needing to have authority over clients. When a client fails to comply with instructions, providers are at times abusive or harsh and justify this treatment as necessary to prevent harm to the client or her child.

#### Client does not follow instructions of provider

Throughout labor, providers ask clients to assume certain positions, to refrain from pushing too early, to push at the right time, or to respond to other instructions. Providers reported that clients often do not follow these instructions and that harsh treatment is often necessary to get them to comply. However, several providers also explained that relatives of the client are often able to get her to follow these instructions which would suggest that the provider lacks the rapport necessary or has not been able to explain in a way that the client understands. In other instances, we observed that the extreme pain clients were experiencing seemed to inhibit their ability to process or respond promptly to a provider’s instructions.

Clients frequently report negative experiences in the delivery room and the pain of labor is extremely salient. In order to expedite labor, many providers and clients remarked that clients consume herbs to hasten delivery. Providers shared their concern for client complications as a result of taking herbs, and we observed providers scolding clients who were dilating abnormally quickly for taking herbs.

When clients fail to push when they are told to, this may be due to fatigue if they did not eat during labor (a common precaution women take to avoid defecating during delivery). Describing this situation, a provider said “Mothers who are difficult don’t want to push … and some say ‘no, I haven’t eaten since three days ago’—'why haven’t you eaten? How are you going to push your baby?’” Clients who have delivered previously might not comply with an instruction that would contradict prior experience. Whether or not this is the case, providers perceive those who have given birth previously as less compliant: according to one provider, “The multigravidas, they’ve gone through that before and most of the time they’re the ones who are not very cooperative.”

In general, providers think that rigid instruction or harsh treatment enhance compliance, and report few other strategies to ensure clients’ cooperation, with the exception of referral to a higher-tier facility. The comments of one provider were representative: “if they don’t listen we usually refer; maybe I don’t have knowledge on [how to handle her case], so what can I do? So I’d just refer her.” Many providers with whom we spoke acknowledged rigid or harsh treatment as a necessary evil reserved for moments when compliance was necessary to keep the client and her child safe. In these cases, providers showed a propensity for explaining their own suboptimal behavior as in service of avoiding a worse outcome.

Providers use the ever-present high stakes to justify this treatment; as one provider put it, “You’ll be having stillbirth all the time if at times you don’t yell at difficult patients.” Not only did providers yell, but even threatened clients with the worst outcomes; one provider described telling clients, “Labor is painful, but you have to accept whatever we are telling you. You shouldn’t do things which you don’t know … Without us telling you, you might have stillbirth.” Some even felt that clients, in retrospect, sometimes appreciated this harsh care because it kept them safe, although our interviews with postpartum women suggest otherwise.

## Discussion

Our research supports the findings of other literature around the importance of ensuring that providers concretize an intention to change the way they provide care in alignment with best practices of respectful care [13, 26–29]. We highlight that initial on-site training, observed behavior of other providers, and ongoing feedback and supervision all emphasize the importance of death avoidance, and either actively support harsh treatment as a means to achieve client cooperation or justify this behavior. In order to change practices in the delivery room, not only must we generate a moment of reflection on current treatment practices, but we must also seek to transform the context in which providers work so that their environment emphasizes the importance of client experience of care as a core function of providers’ work.

Formative research from the behavioral sciences sheds light on the behavioral mechanisms linking specific contextual features with the behavioral barriers described. For instance, our research suggests that the mental model providers have of their role is narrowly focused on clinical functions and death avoidance. Research on the formation of mental models [20] suggests that when formative cues are multi-faceted and the individual has repeated and continuous interaction with these cues, they are likely to shape the way an individual views that concept. In order to reshape the mental model that providers hold of their role and of good care, the different features in their environment which support this mental model must be shifted. Our findings suggest that providers may rely on automaticity [21] to direct their actions during delivery, thus any shift toward a new standard of care must be explicit.

Behavioral research also points to the role of defaults in guiding behavior, especially in situations when individuals may operate from a place of cognitive scarcity and as such are not consciously considering all of their options before acting [19, 30]. For instance, if during the critical learning period of childhood providers were not exposed to other means of correcting behavior, research on availability bias [31] would suggest that providers may in turn view being stern or harsh as the best or even only option for correcting non-compliance, in this case with a client during delivery. Descriptive norms in the facility further reinforce this default as other experienced providers enact harsh behavior and then justify it as a means to avoid client or infant death. In order to promote respectful maternity care, the default means of ‘gaining compliance’ must be shifted by reshaping the cues, including the role of peer influence, in providers’ environment.

Our research also highlights that addressing provider intention to provide respectful care may not be sufficient to guarantee absence of disrespect and abuse. Lack of client cooperation often triggers disrespect and abuse, and at times client behavior does put clients at risk for delivery complications. By understanding the behavioral drivers underlying a client’s failure to follow provider instructions, we are able to unearth additional opportunities for improving client experience and quality of care. Our findings suggest that a client’s negative experience in the delivery room may initiate a vicious cycle which puts clients at higher likelihood of future abuse. For instance, a client who has experienced severe pain with little comfort may be more likely to arrive late to the facility the next time she delivers to allow herself to be in the comfort of her home and family for a longer period of time. She may also be more prone to consume herbs or other remedies to accelerate the process of delivery. Both of these actions complicate the work of the provider and may trigger disrespect and abuse. This experience with harsh treatment further solidifies in the mind of the client her desire to avoid the labor ward until it is absolutely necessary if at all. By improving client experience, especially for first time mothers, there are opportunities to break this cycle of disrespect and abuse.

Several limitations apply to this work. Our interview findings may be subject to bias due to the way questions were asked or the identity of the researcher. Interview guides were vetted for biased or leading questions, and the number of interviews conducted as well as duration of acclimation time may have limited this bias. Because observations occurred at the same two facilities for multiple hours each instance, providers likely became acclimated to our presence and acted in accordance with typical behavior. It is unclear to what extent our findings can be generalized to other settings, though where contextual features are similar, provider behaviors may manifest similarly as well. Because our research was observational rather than experimental, we are unable to definitively assert the presence of certain psychological mechanisms believed to be in play. Where evidence suggests these psychological concepts may apply, it is still reasonable to design solutions with them in mind even in an absence of certainty.

## Conclusions

Improving the experience of care for women during delivery is an issue of critical importance from both health outcomes and human rights perspectives. In recent years, a growing body of research has demonstrated that disrespect and abuse is prevalent around the globe and has identified a range of different factors which impact the behavior of providers. Our research builds on this evidence by identifying the specific features in the environment of providers, whether personal experience, social norms, organizational priorities, among others which inhibit provision of respectful care, drawing from the behavioral science literature on decision-making and follow through. Bringing together insights from multiple disciplines can lead to a more nuanced understanding of this challenging problem and lead to different, complementary solutions which can help transform the experience of delivery for both providers and clients.

## Data Availability

The datasets generated and analyzed during the current study are not publicly available since publicizing them could compromise study participants’ privacy and anonymity, but are available from the corresponding author on reasonable request.

## Abbreviation

SMAG: Safe Motherhood Action Group

## Footnotes

1 Observations took place in urban rather than rural facilities since visits to rural facilities did not coincide with a delivery, due to lower client volumes. Disrespectful care also appeared less pervasive in rural facilities, suggesting a focus on urban facilities was warranted.
